# 

**Published:** 1855-01

**Authors:** 


### THE Dental Register of the West.

JAMES TAYLOR, M. D., D. D. S., Editor.

Published Quarterly at $2 per Annum.

CINCINNATI:
T. WRIGHTSON & CO., PRINTERS,
167 Walnut Street.

